# Lysine-specific demethylase 1 (LSD1) destabilizes p62 and inhibits autophagy in gynecologic malignancies

**DOI:** 10.18632/oncotarget.20158

**Published:** 2017-08-10

**Authors:** Angel Chao, Chiao-Yun Lin, An-Ning Chao, Chia-Lung Tsai, Ming-Yu Chen, Li-Yu Lee, Ting-Chang Chang, Tzu-Hao Wang, Chyong-Huey Lai, Hsin-Shih Wang

**Affiliations:** ^1^ Department of Obstetrics and Gynecology, Chang Gung Memorial Hospital and Chang Gung University, Taoyuan, Taiwan; ^2^ Gynecologic Cancer Research Center, Chang Gung Memorial Hospital, Kaohsiung, Taiwan; ^3^ Department of Ophthalmology, Chang Gung Memorial Hospital and Chang Gung University, Taoyuan, Taiwan; ^4^ Genomic Medicine Research Core Laboratory, Chang Gung Memorial Hospital, Taoyuan, Taiwan; ^5^ Department of Pathology, Chang Gung Memorial Hospital and Chang Gung University, Taoyuan, Taiwan

**Keywords:** gynecologic malignancies, LSD1, autophagy, p62

## Abstract

Lysine-specific demethylase 1 (LSD1) – also known as KDM1A – is the first identified histone demethylase. LSD1 is highly expressed in numerous human malignancies and has recently emerged as a target for anticancer drugs. Owing to the presence of several functional domains, we speculated that LSD1 could have additional functions other than histone demethylation. P62 – also termed sequestasome 1 (SQSTM1) – plays a key role in malignant transformation, apoptosis, and autophagy. Here, we show that a high LSD1 expression promotes tumorigenesis in gynecologic malignancies. Notably, LSD1 inhibition with either siRNA or pharmacological agents activates autophagy. Mechanistically, LSD1 decreases p62 protein stability in a demethylation-independent manner. Inhibition of LSD1 reduces both tumor growth and p62 protein degradation *in vivo*. The combination of LSD1 inhibition and p62 knockdown exerts additive anticancer effects. We conclude that LSD1 destabilizes p62 and inhibits autophagy in gynecologic cancers. LSD1 inhibition reduces malignant cell growth and activates autophagy. The combinations of LSD1 inhibition and autophagy blockade display additive inhibitory effect on cancer cell viability. A better understanding of the role played by p62 will shed more light on the anticancer effects of LSD1 inhibitors.

## INTRODUCTION

Methylation is a form of post-translational covalent modification of histones that epigenetically regulates specific gene expression patterns. Lysine-specific demethylase 1 (LSD1; also known as KDM1A; Gene ID 23028) – the first identified histone demethylase – is a monoamine oxidase (MAO) homologue that demethylates mono- or dimethylated histone H3 lysine 4 (H3K4) and H3 lysine 9 (H3K9) through amine oxidation [1].

As a flavin adenine dinucleotide (FAD)-dependent enzyme, LSD1 consists of three major domains − an N-terminal SWIRM domain, a central protruding tower domain, and a C-terminal amine oxidase like (AOL) domain [2]. Growing evidence indicates that LSD1 is critical for human tumorigenesis, and its expression is increased in several malignancies – including bladder cancer [3], prostate cancer [4], non-small cell lung cancer [5], breast cancer [6, 7], colon cancer [8], uterine endometrioid adenocarcinoma [9, 10], as well as ovarian serous and mucinous adenocarcinomas [11–14]. Epidermal growth factor has been shown to stimulate LSD1 expression [13], which in turn promotes epithelial-mesenchymal transition [14]. Owing to the presence of several functional domains, we speculate that LSD1 could have additional functions other than histone demethylation.

Because of its involvement in malignant cell proliferation, migration, and invasiveness [3, 5, 8, 13, 14], pharmacological inhibition of LSD1 holds promise as a novel anticancer strategy. Currently available LSD inhibitors can be classified into three categories: (i) MAO-A/B inactivators (e.g., pargylin, tranylcypromine [TCP], phenelzine), (ii) peptide-based inhibitors, and (iii) polyamine-based inhibitors [2]. SP2509 is a polyamine-based, highly potent, specific, and reversible LSD1 inhibitor that acts as a non-MAO-A/B inactivator [15]. SP2509 inhibits tumor cell proliferation in solid malignancies (e.g., Ewing sarcoma and colorectal, breast, and endometrial cancers) [10, 15, 16], as well as in acute myeloid leukemia (AML) [17]. In the latter case, SP2509 inhibits the proliferation of AML blasts synergically with the pan-histone deacetylase inhibitor panobinostat [17].

Autophagy – one of the cellular mechanisms to maintain metabolic homeostasis – plays a controversial role in cancer biology, either exerting a prosurvival or an antiproliferative effect [18]. For example, chemotherapeutic agents induce cellular and metabolic stress that activates autophagy as a prosurvival factor (ultimately delaying apoptotic cell death and promoting both tumor progression and chemoresistance) [19–21]. In contrast, autophagy has been shown to suppress tumorigenesis [22], and autophagy activation can exert antitumoral effects [23].

The autophagic process begins with the formation of autophagosomes, followed by their fusion with lysosomes to form autolysosomes (the degradative form of autophagic vacuoles) which finally undergo self-digestion [24, 25]. P62 – also termed sequestasome 1 (SQSTM1) – is a key component of autophagic machinery [26]. By virtue of its different functional domains, p62 is capable of interacting with different cellular signaling proteins (e.g., MEK, ERK, RIP, TRAF6) and the autophagic protein LC3 [27]. Therefore, p62 plays a key role at the crossroads of cancer, apoptosis, and autophagy [28].

In the field of gynecologic malignancies, advanced-stage ovarian cancer is characterized by a lower autophagic activity compared to early-stage tumors [29]. LSD1 has been associated with autophagy. For instance, double knockdown of LSD1 and ubiquitin factor E4B activate autophagy and proteasomal activity [30]. The LSD1 inhibitor NCL1 was shown to promote prostate cancer cell death via induction of autophagy [4], suggesting that LSD1 may contribute to the control of autophagic flux in malignant cells. Another LSD1 inhibitor S2101 inhibited ovarian cancer cells via apoptosis and autophagy [31]. However, the mechanisms by which autophagy is regulated in LSD1-overexpressing gynecologic malignancies remain largely unclear.

We therefore designed the current study with the two goals: (i) to examine whether LSD1 is overexpressed in other gynecologic malignancies, including multiple ovarian cancer types and uterine serous carcinoma (USC; a clinically aggressive subtype of endometrial cancer); and (ii) to investigate the effect of LSD1 inhibition on gynecologic tumor growth in relation to changes in the autophagic flux. Our main findings indicated that LSD1 destabilizes the autophagy substrate p62.

## RESULTS

### Elevated LSD1 promotes tumorigenesis in gynecologic cancer

**Figure 1:**
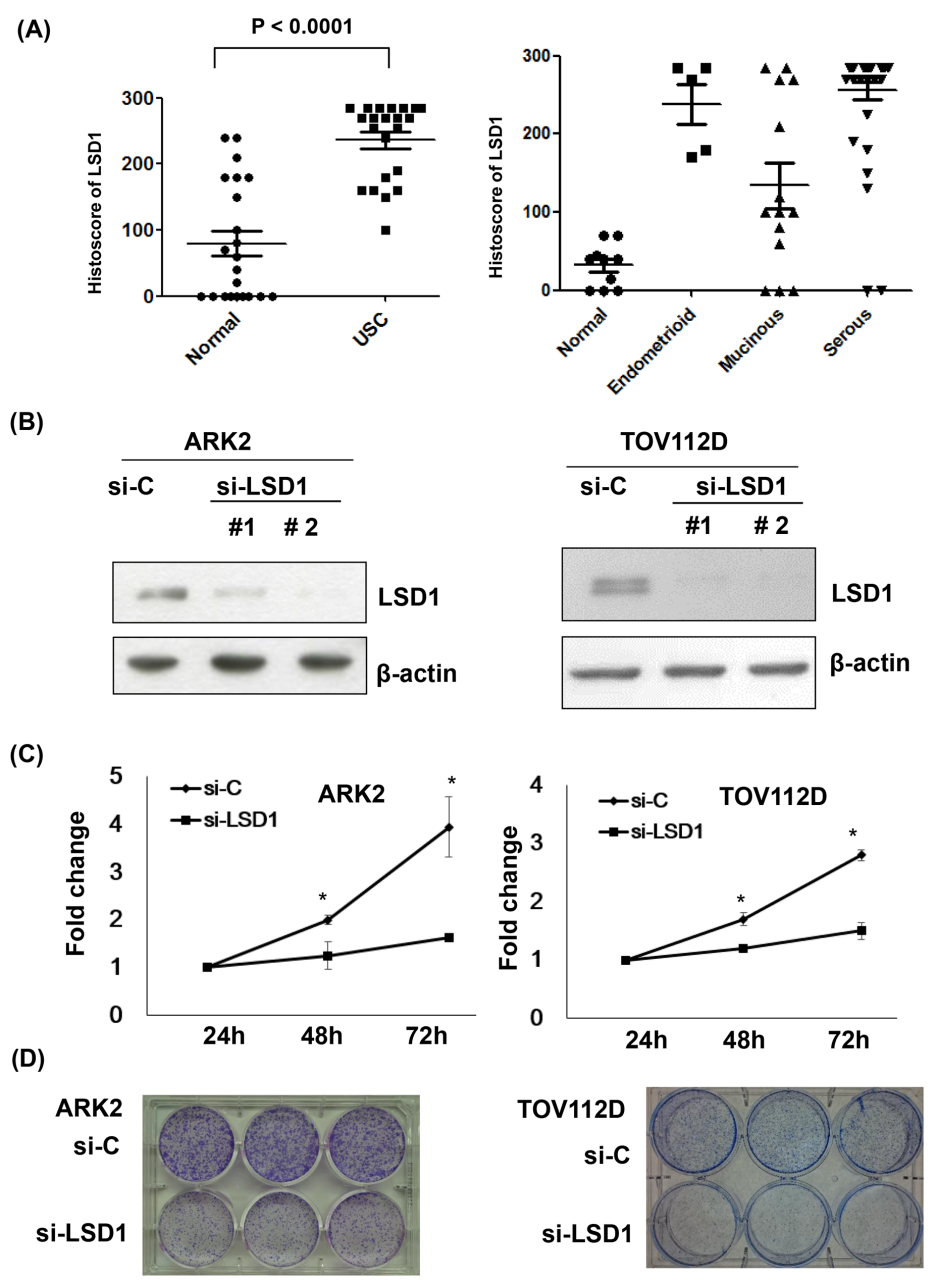
Increased LSD1 expression in gynecologic malignancies **(A)** LSD1 immunostaining intensities were analyzed using histoscores (calculated as the percentage of LSD1-positive cells multiplied by their staining intensity). Histoscores were determined in uterine serous carcinoma (USC) samples obtained from primary surgery (n = 22) as well as in adjacent normal tissues (Normal). Tissue arrays of different histological ovarian cancer (OVCA) types were also examined (serous carcinoma, n = 41; endometrioid carcinoma, n = 5; mucinous carcinoma, n = 14; normal ovarian tissue, n = 10). **(B)** LSD1 expression was knocked down with siRNA. Uterine serous carcinoma ARK2 cells and ovarian cancer TOV112D cells were harvested after exposure to control siRNA (si-C), LSD1 siRNA #1, or LSD1 siRNA #2 for 72 h. Equal amounts of whole-cell extracts were subjected to immunoblots with antibodies raised against LSD1 or β-actin. **(C)** Knocking down p62 resulted in a significant time-dependent decrease in cell proliferation. Data are expressed as means ± standard errors from three independent experiments. * P < 0.05 compared to control. **(D)** LSD1 siRNA inhibited colony formation.

LSD1 histoscores of ovarian and endometrial cancer tissue arrays revealed that LSD1 protein levels were higher in tumors compared to the surrounding normal tissues (Figure 1A; P < 0.001). Treatment of uterine serous carcinoma ARK2 cells and ovarian cancer TOV112D cells with two different LSD1 siRNAs (#1 and #2) significantly reduced LSD1 protein expression (-75% and -90%, respectively; Figure 1B and Supplementary Figure 1). Because LSD1 siRNA #2 resulted in a more marked inhibition, all subsequent silencing experiments were based on its use. Both cell proliferation (Figure 1C) and colony formation (Figure 1D) were significantly reduced by LSD1 siRNA #2 treatment, suggesting that LSD1 promotes gynecologic cancer tumorigenesis.

### Inhibition of LSD1 activates autophagy

**Figure 2:**
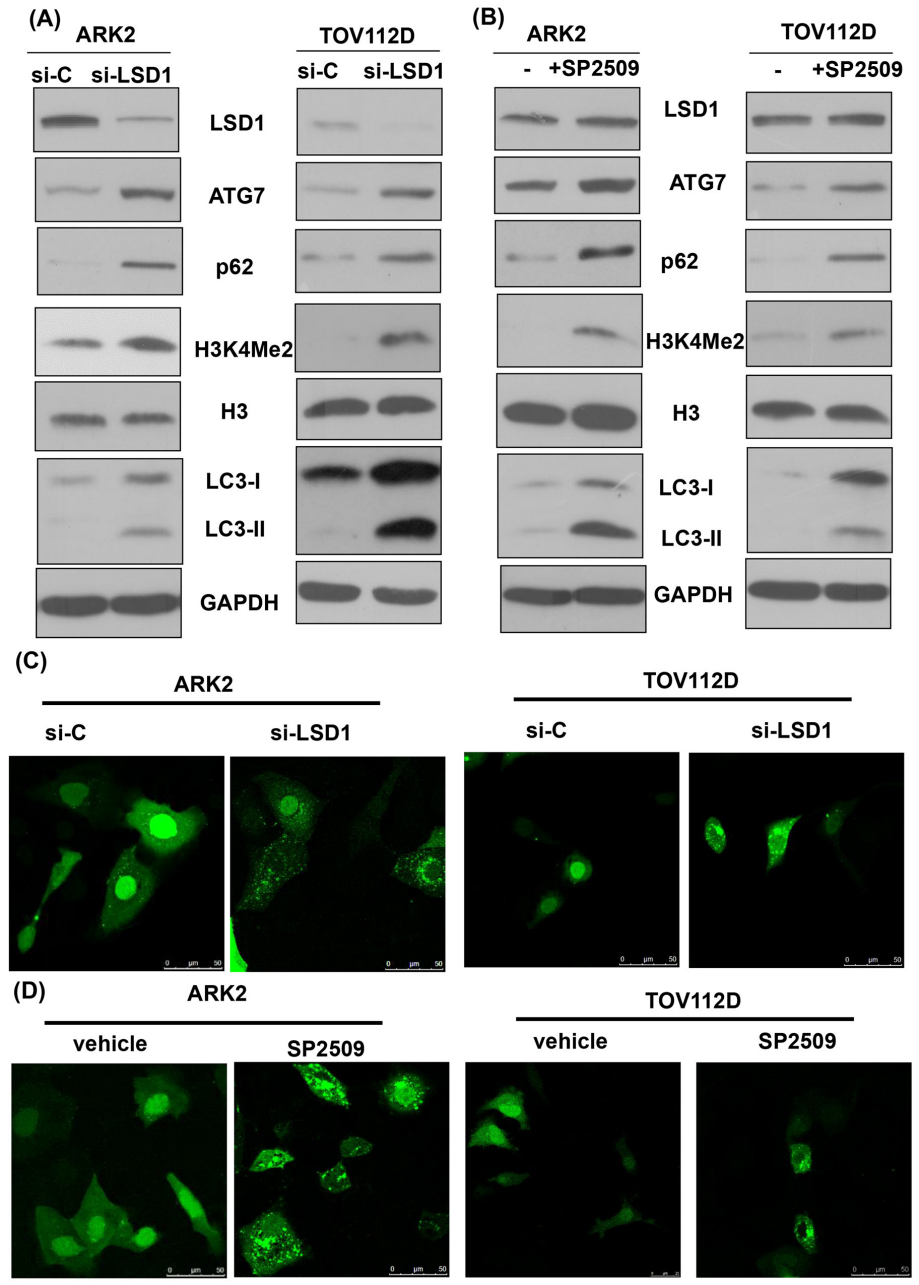
Inhibition of LSD1 activates autophagy **(A)** Uterine serous carcinoma ARK2 cells and ovarian cancer TOV112D cells were transiently transfected with control siRNA (si-C) or LSD1 siRNA #2 for 72 h. Cell lysates were subsequently subjected to western blots. **(B)** ARK2 and TOV112D cells were treated with an LSD1 inhibitor SP2509 (100 nM) for 24 h. Equal amounts of protein lysates were analyzed with western blots with appropriate antibodies. Increased levels of H3K4Me2 indicated an inhibition of LSD1. GAPDH was used to confirm that equal amounts of proteins were present in all lanes. **(C)** The formation of immunofluorescent puncta structures was observed in ARK2 and TOV112D cells transfected with GFP-LC3 followed by transfection with si-C or LSD1 siRNA #2 for 72 h. **(D)**The formation of immunofluorescent puncta structures was observed in ARK2 and TOV112D cells transfected with GFP-LC3 followed by treatment with an LSD1 inhibitor SP2509 for 24 h. Quantitation was performed by calculating the ratio of cells with GFP-LC3 dots in multiple visual fields (as shown in Supplementary Figure 2).

Inhibition of LSD1 with either siRNA or the LSD1 inhibitor SP2509 stimulated expression levels of the autophagy markers ATG7 and LC3-II in different cancer cells (Figures 2A and 2B, and Supplementary Figure 2). The detection of increased levels of H3K4Me2 confirmed the functional suppression of LSD1 through siRNA [32]. Fluorescent microscopic detection of puncta formation also indicated that inhibition of LSD1 with either siRNA or the LSD1 inhibitor SP2509 stimulated autophagy (Figures 2C and 2D, and Supplementary Figure 3). Of note, inhibition of LSD1 with either siRNA or the LSD1 inhibitor SP2509 increased p62 levels (Figure 2A and 2B, and Supplementary Figure 2). Because p62 is digested in autophagosomes, its decrease indicates a completed autophagic process [24]. Our seemingly contradictory findings of increased p62 levels (Figure 2A and 2B) and autophagy activation (Figure 2C and 2D) prompted us to investigate further the mechanistic interactions between LSD1 and p62.

### LSD1 binds to p62 but does not demethylate p62

**Figure 3:**
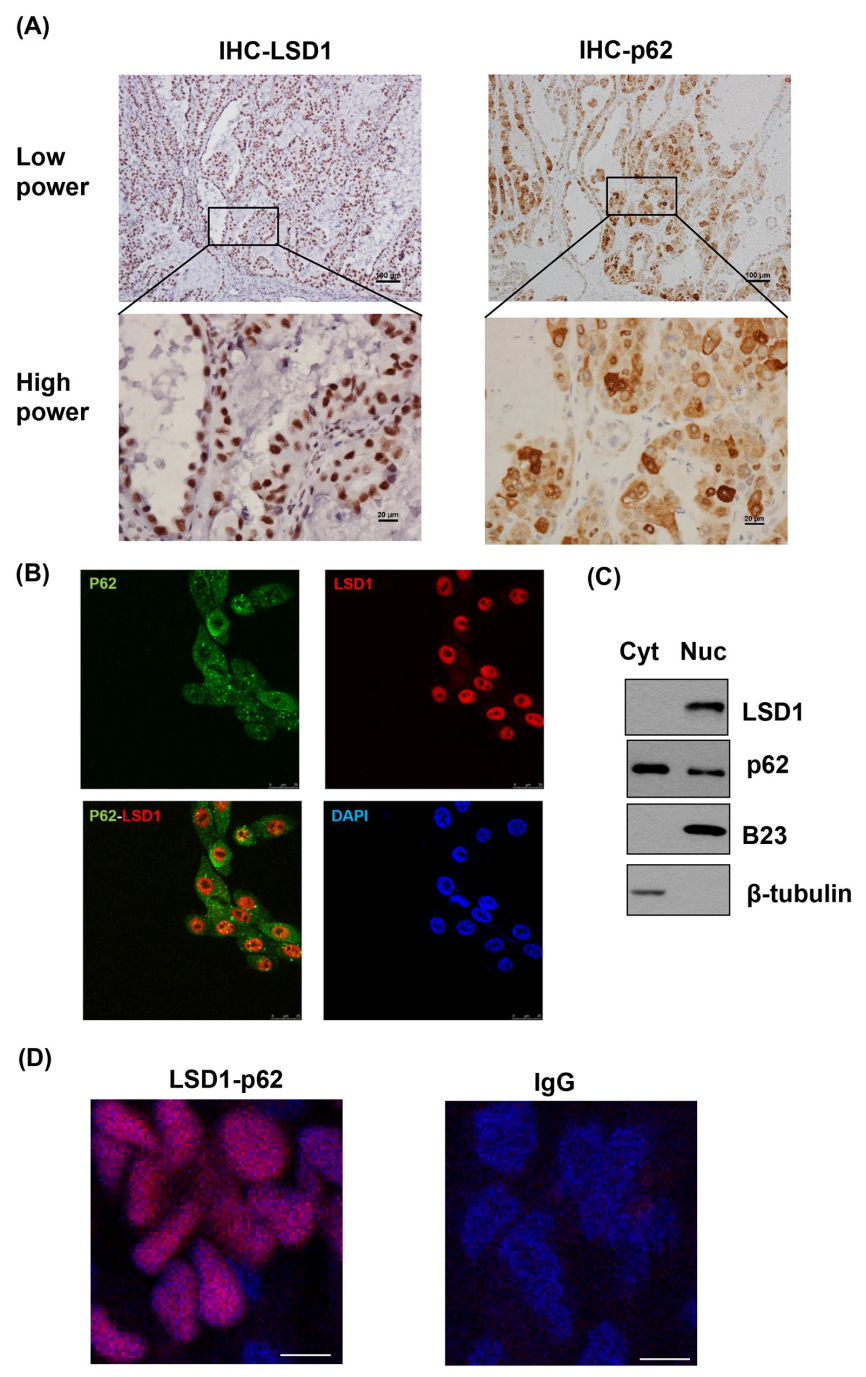

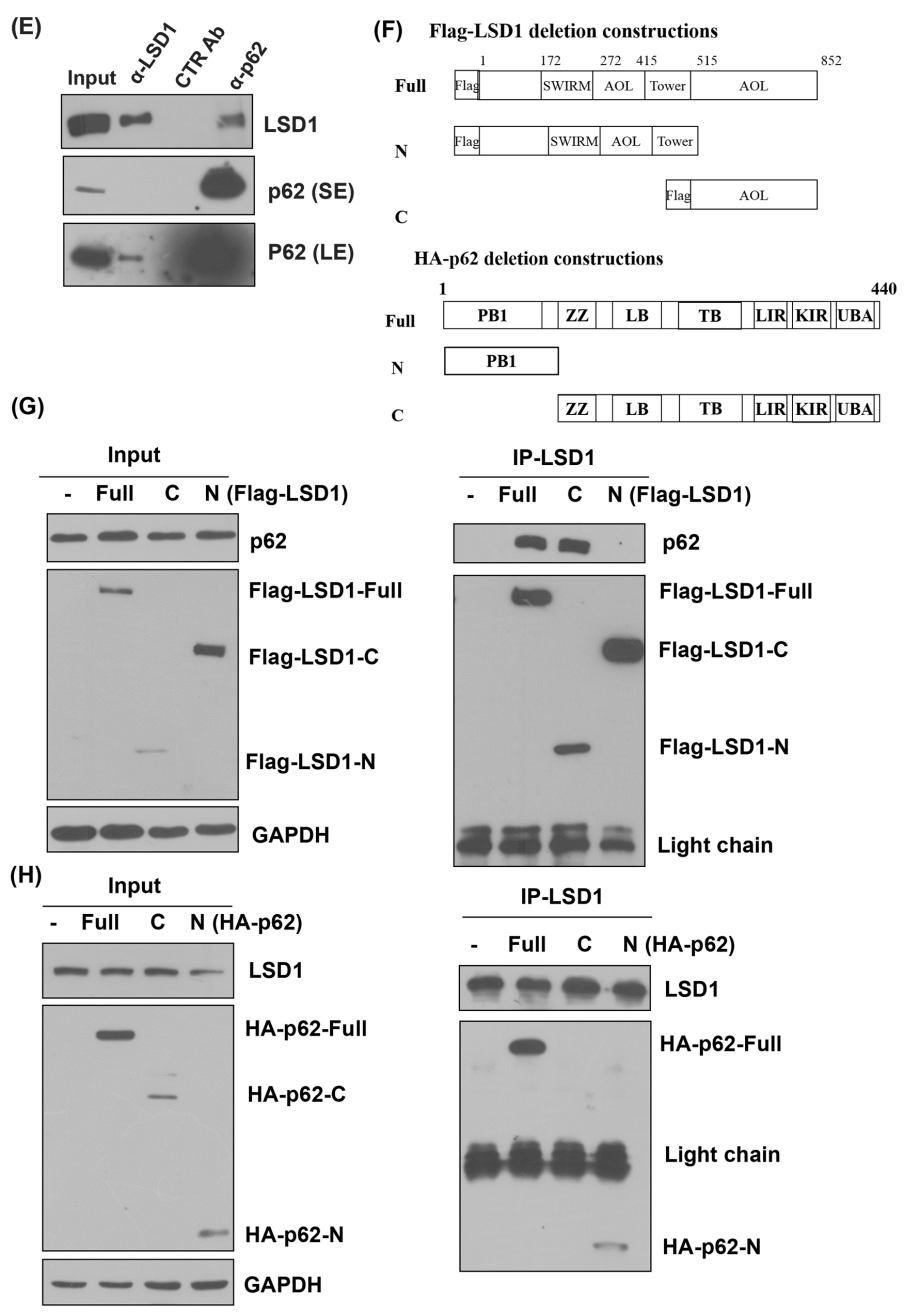
Colocalization and interaction between LSD1 and p62 **(A)** Representative immunohistochemical staining showing the co-localization of LSD1 (left panel) and p62 (right panel) in ovarian cancer cells. P62 expression is localized in the cytoplasm, late endosomes, and the nucleus. Notably, both p62 and LSD1 were co-localized in the nucleus. **(B)** Immunofluorescence confocal microscopy was used to localize LSD1 (red) and p62 (green). Nuclei were stained in blue (DAPI). Scale bars indicate 25 μm. **(C)** Subcellular fractionation of uterine serous carcinoma ARK2 cells was used to analyze LSD1 and p62 in different subcellular compartments. β-tubulin and B23 were used as markers for the cytoplasm (Cyt) and nuclear (Nuc) fractions, respectively. **(D)** A proximity ligation assay (PLA) using anti-LSD1 and anti-p62 antibodies was performed to confirm the interaction between LSD1 and p62 in ovarian cancer tissues (left panel). An IgG was used as a negative control for the first antibody (right panel). Nuclei were stained in blue (DAPI). Scale bars indicate 5 μm.**(E)** ARK2 whole-cell lysates were immunoprecipitated (IP) with an anti-LSD1 (α-LSD1) or anti-p62 (α-p62) antibody and subsequently analyzed by immunoblotting with an anti-p62 antibody or anti-LSD1 antibody. A control IgG antibody (CTR Ab) was used for mock immunoprecipitation. For the α-LSD1 pulldown, although the p62 protein did not appear in the short exposure (SE) of western blot, the p62 band was very clear in the long exposure (LE) of the same blot. **(F)** Upper panel: LSD1 structure with the chromatin factor-associated SWIRM (SWI3, RSC8, and Moira) domain, the amine oxidase-like (AOL) domain, and the LSD1 tower domain (TOWER). Lower panel: p62 structure with the Phox and Bem1p (PB1) domain, the zinc finger (ZZ) Rip 1 binding domain, the LIM protein Ajuba binding domain (LB), the TRAF6-binding domain (TB), the LC3-interacting region (LIR), the Keap1-interacting region (KIR), and the ubiquitin-associated domain (UBA). **(G)** Lysates from ARK2 cells transiently overexpressing Flag-tagged LSD1 (FL, N, or C) were immunoprecipitated with an anti-LSD1 antibody and subsequently subjected to immunoblotting with antibodies raised against Flag, p62, or GAPDH. GAPDH was used to confirm equal protein inputs in all lanes. **(H)** Lysates from ARK2 cells transiently overexpressing HA-tagged p62 (FL, N, or C) were immunoprecipitated with an anti-LSD1 antibody and subsequently subjected to immunoblotting with antibodies raised against HA, LSD1, or GAPDH. GAPDH was used to confirm equal protein inputs in all lanes.

Both LSD1 and p62 were stained in the nucleus of cancer tissues (Figure 3A). Immunofluorescent microscopy showed that LSD1 was predominantly localized in the nucleus, whereas p62 was expressed in the cytoplasm, late endosomes, and the nucleus. Notably, both p62 and LSD1 were co-localized in the nucleus (Figure 3B). Subcellular fractionation analyses also supported that LSD1 and p62 were co-expressed in the nucleus (Figure 3C). Proximity ligation assay (PLA) indicated the interaction between LSD1 and p62 in the nucleus of cancer cells (Figure 3D), a finding validated by co-immunoprecipitation of LSD1 and p62 (Figure 3E). Furthermore, immunoprecipitation experiments using LSD1 deletion constructs demonstrated that LSD1 interacted with p62 via its C-terminal AOL domain (Figure 3F and 3G). The use of different p62 deletion constructs also showed that the N-terminal PB1 domain of p62 interacted with LSD1 (Figure 3F and 3H). Collectively, these results indicated a biochemical interaction between the C-terminal AOL domain of LSD1 and the N-terminal PB1 domain of p62.

We also tested whether LSD1 can demethylate p62. To this aim, LSD1 was initially knocked down through RNA silencing. Endogenous p62 was subsequently immunoprecipitated with an anti-p62 antibody, and the total methylated lysine signal was analyzed with western blot. We used p53 as a positive control for the LSD1 substrate (Supplementary Figure 4A) [33]. However, the results indicated that LSD1 was unable to demethylate p62 (Supplementary Figure 4B).

### LSD1 decreases p62 protein stability

**Figure 4:**
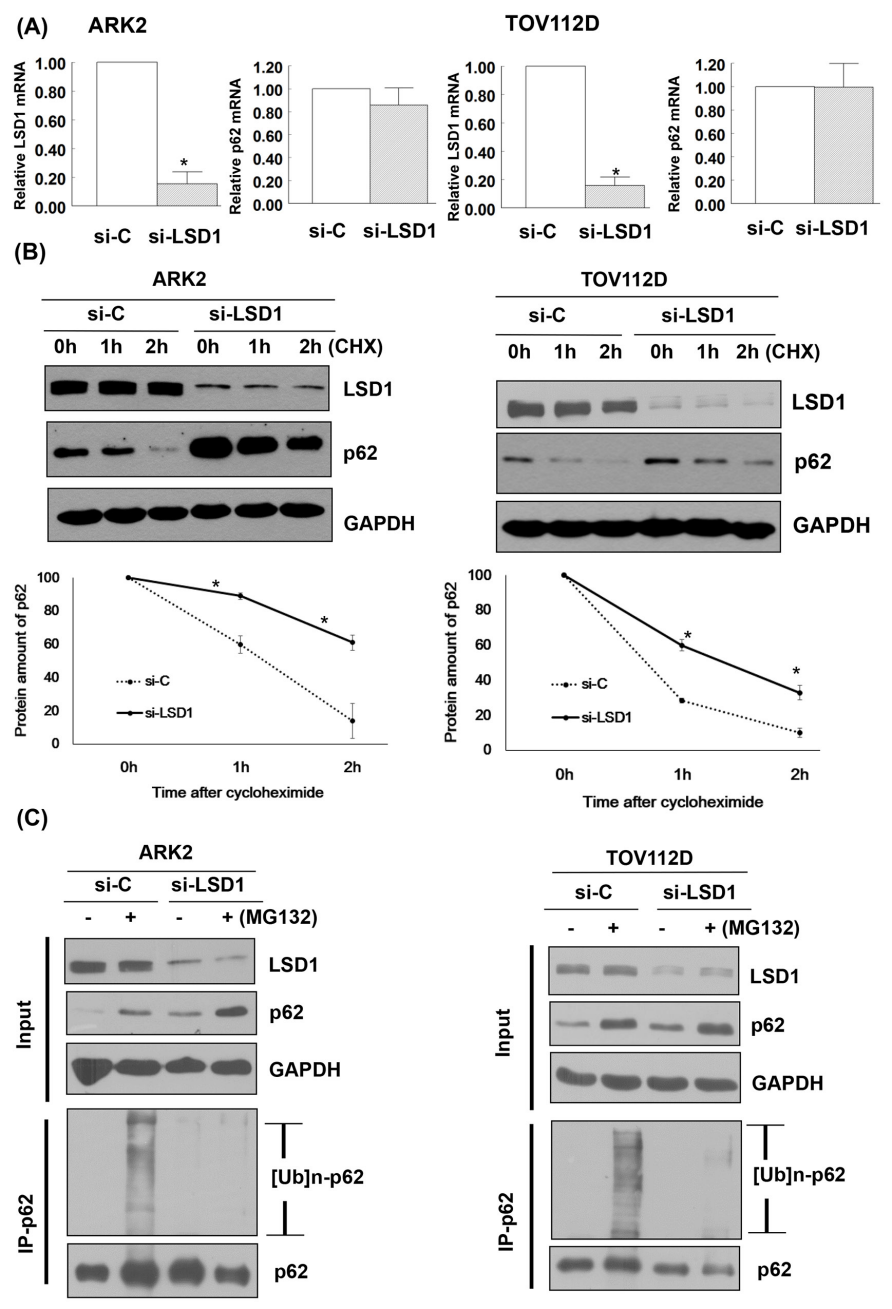
LSD1 decreases p62 protein stability **(A)** Uterine serous carcinoma ARK2 cells and ovarian cancer TOV112D cells were transiently transfected with control siRNA (si-C) or LSD1 siRNA #2 for 72 h; mRNA expression was subsequently analyzed with real-time QPCR. Data in bar charts are expressed as means ± standard errors of the mean. **(B)** ARK2 and TOV112D cells were transiently transfected with si-C or LSD1 siRNA #2 for 72 h and treated with CHX (25 μg/mL). Cell lysates were prepared at the designated time points. Western blot was performed using LSD1, p62, and GAPDH antibodies. Lower panels: the amount of p62 protein measured at each time point was normalized to p62 expression levels at baseline. Data are expressed as means ± standard errors of the mean from three independent experiments. **(C)** ARK2 and TOV112D cells were treated with si-C or LSD1 siRNA #2 for 72 h followed by MG132 (10 μM) for 24 h. Whole-cell lysates prepared in WCE lysis buffer were immunoblotted and immunoprecipitated with a mouse monoclonal antibody directed against p62. Immunocomplexes were probed with antibodies raised against ubiquitin (Ub) and p62.

LSD1 knockdown did not affect p62 mRNA expression (Figure 4A). We then examined whether LSD1 can regulate p62 stability. Our results revealed that LSD1 knockdown stabilized p62 in a time-dependent manner. When new protein synthesis was blocked by treatment with the translational inhibitor cycloheximide (CHX), LSD1 depletion resulted in reduced p62 protein degradation (Figure 4B). To investigate whether the ubiquitination and proteasomal degradation of p62 mediates the observed effects of LSD1 on p62 stability, we treated LSD1-depleted ARK2 cells with the proteasome inhibitor MG132. Treatment with MG132 resulted in increased p62 protein levels, suggesting that proteasomal mechanisms govern p62 stability (upper panel, Figure 4C). Knockdown of LSD1 decreased ubiquitin-conjugated p62 levels (lower panel, Figure 4C), indicating that LSD1 is required for p62 ubiquitination.

### Inhibition of LSD1 reduces tumor growth and p62 protein degradation *in vivo*

**Figure 5:**
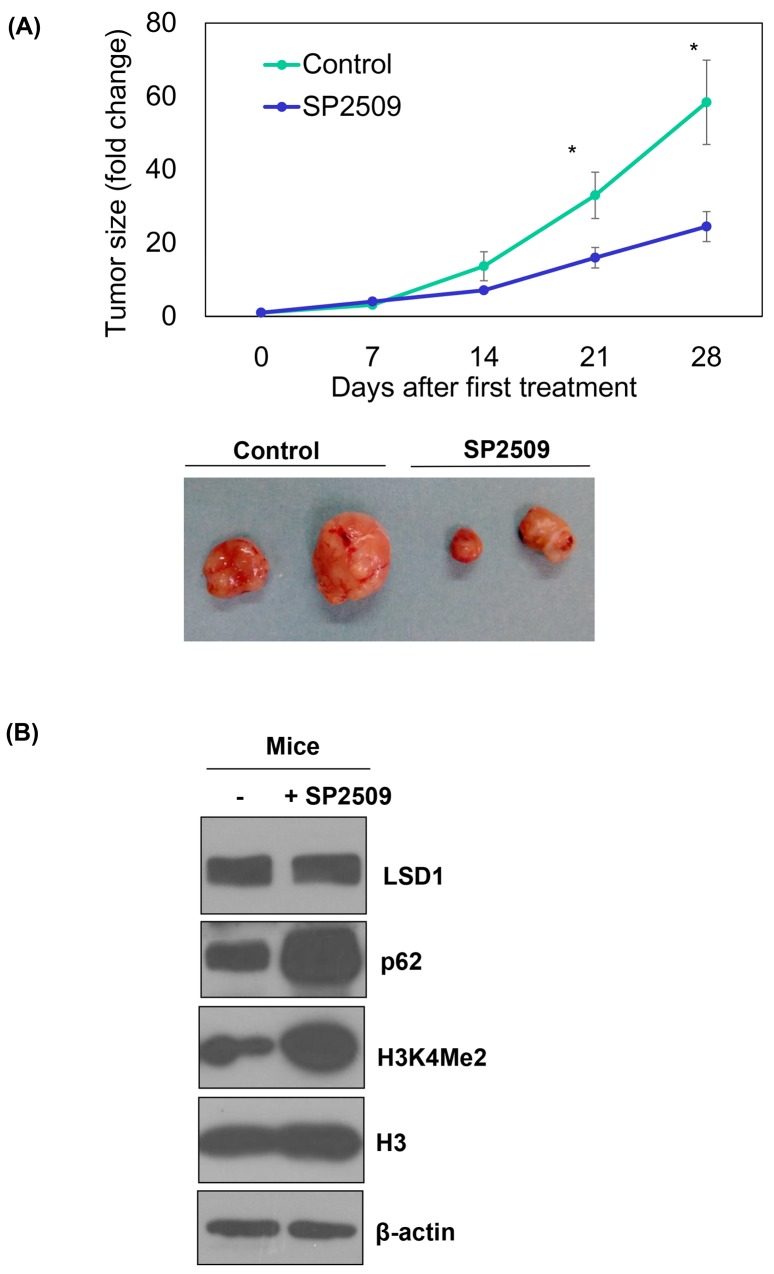
*In vivo* regulation of p62 by an LSD1 inhibitor Uterine serous carcinoma ARK2 cells were subcutaneously injected into the lateral hind leg of nude mice. Xenografted tumors were treated with subcutaneous injections of SP2509 or a vehicle for 4 weeks. **(A)** Tumor diameter was measured weekly and tumor volumes (cm^3^) were calculated. * P < 0.05 compared to the SP2509 group. Representative tumors were taken from tumor-bearing nude mice treated with SP2509 or a vehicle. **(B)** Tumors treated with SP2509 or a vehicle were immunoblotted with the designated antibodies. b-actin was used to confirm equal protein input in all lanes.

Treatment with the LSD1 inhibitor SP2506 of nude mice with xenografted ARK2 cells significantly reduced tumor growth *in vivo* (P < 0.05; Figure 5A), although malignancies were not completed cleared. The analysis of xenografted tumor tissues confirmed that SP2509 inhibited LSD1 activity (as reflected by increased H3K4Me2 levels; Figure 5B). However, elevated p62 levels casted doubts on the clinical usefulness of this approach because we [34] and others [25] have previously shown a role for autophagy activation in tumor resistance to anti-cancer therapy.

### LSD1 inhibition and p62 knockdown promote cancer cell death in an additive manner

**Figure 6:**
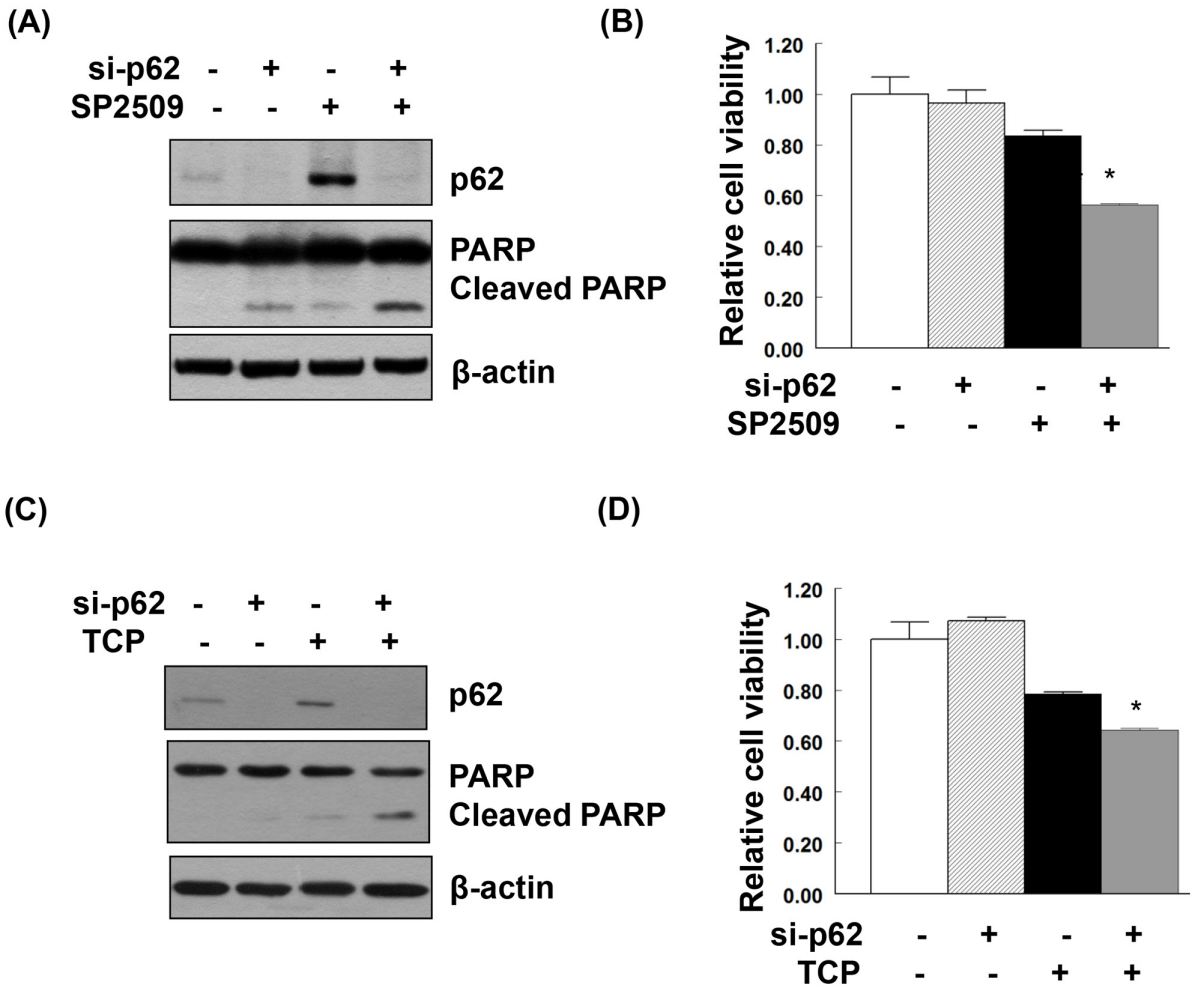
Additive effect on apoptosis and suppression of cell viability induced by the combination of LSD1 inhibitors and p62 knockdown Uterine serous carcinoma ARK2 cells were transiently transfected with si-C or p62 siRNA (si-p62) for 48 h and subsequently treated with 100nM SP2509 **(A, B)** or 100μM TCP **(C, D)** for 24 h. Equal amounts of protein lysates were subjected to immunoblotting with the indicated antibodies. β-actin was used to confirm equal protein inputs in all lanes. **(B, D)** Cell survival was analyzed with MTT assays.

Treatment of uterine serous carcinoma ARK2 cells with the LSD1 inhibitor SP2509 induced apoptosis. When p62 was knocked down in SP2509-exposed cells, a synergistic effect on cancer cell death was observed (Figure 6A and 6B). Treatment with TCP (a different LSD1 inhibitor) also stimulated LC3 and p62 expression (Supplementary Figure 5). Similarly, TCP-induced cancer cell death was magnified by p62 knockdown (Figure 6C and 6D).

### Synergistic effect of a LSD1 inhibitor and an autophagic inhibitor on cancer cell death

**Figure 7:**
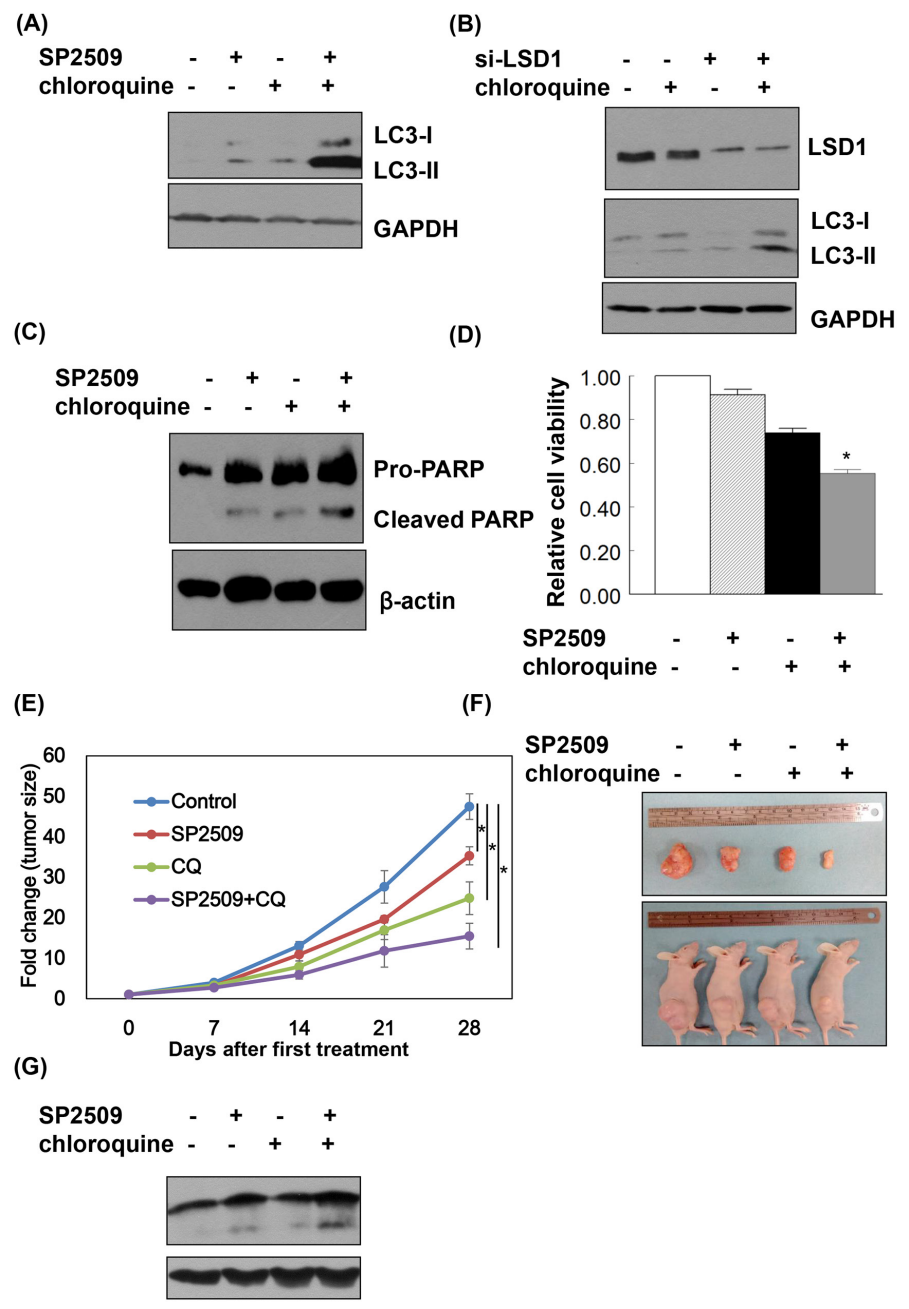
LSD1 inhibition and autophagy blockade exert synergistic effects on cancer cell apoptosis **(A)** Uterine serous carcinomaARK2 cells were treated with 100nM SP2509 and 25nM chloroquine for 24 h. **(B)** ARK2 cells were transiently transfected with si-C or LSD1 siRNA (si-LSD1) for 48 h and subsequently treated with 25nM chloroquine for 24 h. Equal amount of protein lysates were subjected to immunoblotting with the indicated antibodies. GAPDH was used to confirm equal protein inputs in all lanes. **(C)** ARK2 cells were treated with 100nM SP2509 and 25nM chloroquine for 72 h Equal amount of protein lysates were subjected to immunoblotting with the indicated antibodies. β-actin was used to confirm equal protein inputs in all lanes. **(D)** Cell survival was analyzed with MTT assays. **(E)** ARK2 cells were subcutaneously injected into the lateral hind leg of nude mice. Xenografted tumors were treated with subcutaneous injections of vehicle (n = 4), SP2509 (n = 4), chloroquine (CQ) (n = 4), or the combination of SP2509 and chloroquine (SP2509+CQ) (n = 4) for 4 weeks. Tumor diameter was measured weekly and tumor volume (cm^3^) was calculated. * P < 0.05 compared to the control group. **(F)** Representative tumors were taken from tumor-bearing nude mice treated with vehicle, SP2509, CQ or SP2509 +CQ. **(G)** Tumors treated with vehicle, SP2509, CQ or SP2509 +CQ were immunoblotted with the indicated antibodies. GAPDH was used to confirm equal protein input in all lanes.

When cancer cells were treated with a combination of SP2509 and chloroquine, protein levels of LC3-II were higher than in cells treated with SP2509-alone and chloroquine-alone (Figure 7A and 7B). The combination of SP2509 and chloroquine had synergistic inhibitory effects on proliferation via caspase dependent apoptosis (Figure 7C and 7D). Synergistic effect of SP2509 and chloroquine in cancer cells was also observed in the animal model with xenografted tumor (Figure 7E-7G).

## DISCUSSION

**Figure 8:**
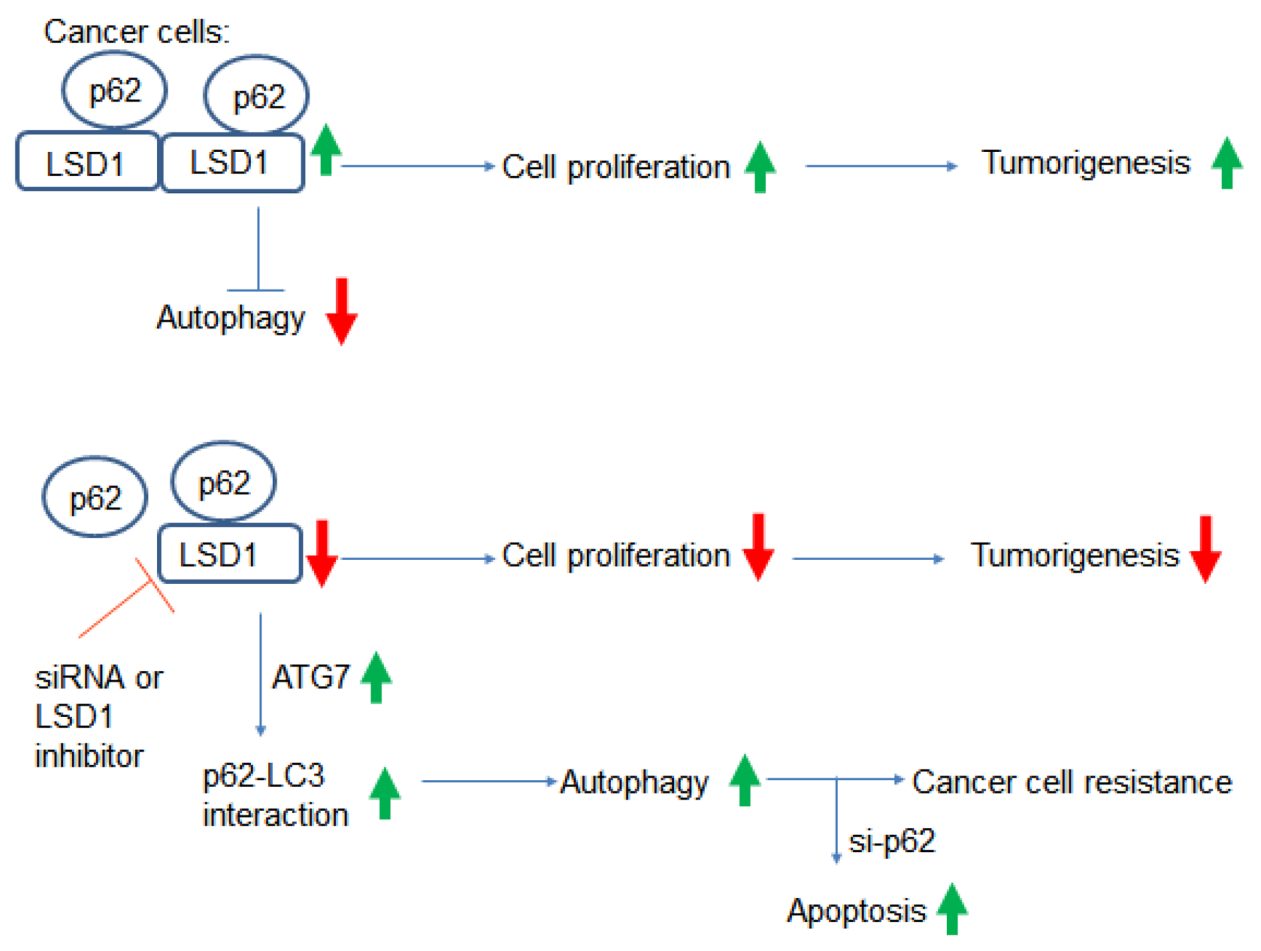
Summary of the interactions between LSD1 and p62 Increased LSD1 expression in cancer cells destabilizes the p62 protein. This may result in a reduced interaction between p62 and LC3, which ultimately suppresses autophagy and promotes tumorigenesis. Inhibition of LSD1 either by RNA silencing or LSD1 inhibitors suppresses cell proliferation, increases p62 levels, and activates autophagy. Although autophagy activation may promote cancer resistance to chemotherapy, this can be overcome by a combination of LSD1 inhibitors and p62 silencing.

To our knowledge, this study is the first to demonstrate a direct interaction between LSD1 and p62. Specifically, our results indicate that LSD1 is capable of interacting and stabilizing the selective autophagy substrate p62 (Figure 8). Suppression of LSD1 with either RNA silencing or pharmacological LSD1 inhibitors decreased cancer cell growth but also activated autophagy (as reflected by increased p62 levels). Taken together, these findings indicate that 1) LSD1 is one of the key molecular player in gynecologic tumorigenesis and 2) LSD1 is directly involved in the regulation of autophagic flux in ovarian cancer and USC cells. Our results may pave the way for developing novel therapeutic strategies based on the combination of LSD1 inhibitors and si-p62 in gynecologic malignancies.

The estrogen-independent endometrial cancer USC has been linked to molecular aberrations in the p53, cyclin E-FBXW7, and PI3K pathways [35]. USC is characterized by poor outcomes and chemotherapy resistance (especially in cases with recurrent disease and/or advanced stages). Our current data suggest that the USC malignant phenotype could at least in part be driven by LSD1 overexpression. Accordingly, LSD1 inhibition (either through RNA silencing or the LSD1 inhibitor SP2509) reduced malignant cell proliferation and colony formation. An intriguing observation from our study is that LSD1 inhibition was accompanied by autophagy activation, a phenomenon which has been related to resistance to cancer therapy.

P62 plays a key role at the crossroads of autophagy, apoptosis, and cancer [25, 27, 28]. Notably, p62 is known to regulate nuclear factor (erythroid-derived 2)-like 2 (NRF2), mTOR, and NF-kB, which are paramount for cancer cell survival [28]. In transformed mouse embryonic fibroblasts and mammary tumor cells, p62 and autophagy act in a synergistic manner to promote cancer cell growth [36]. In a mouse model, increased p62 was shown to be required for survival of Ras-induced lung adenocarcinomas [37]. Changes in p62 levels are commonly utilized as a marker for modifications in autophagic activity [24], with increased p62 being considered as a proxy on an impaired autophagic flux [22]. Although we previously attributed the same significance to increased p62 levels [34], we believe that higher p62 levels elicited by LSD1 inhibition in the current study could reflect distinct cellular events. Because of its versatile biological roles [25, 28], p62 has indeed emerged as a target for anticancer drugs [26, 38, 39].

The LSD1 inhibitor NCL1 (which is not commercially available in Taiwan) has been shown to induce apoptosis and autophagy in prostate cancer cells [4]. Although p62 was not studied in their study [4], Etani *et al.* clearly showed that a combination of NCL1 with the autophagy inhibitor chloroquine inhibited cell growth in an additive manner [4]. Echoing these findings, we demonstrate here that LSD1 inhibition (either with siRNA or pharmacological LSD1 inhibitors) induced both cell death and autophagy. The additive suppression of cell viability by LSD1 inhibitors and p62 siRNA (shown in Figure 6) also points to a critical role of p62 when LSD1 inhibitors are used for anticancer therapy. The question as to whether this approach could be useful to overcome chemoresistance in gynecologic cancers deserves further scrutiny.

Our findings have some limitations. First, animal results to support the synergistic anticancer effect of combined LSD1 and p62 inhibition *in vivo* were not available. In this scenario, the effect of the autophagy inhibitor verteporfin [39] should be investigated in future studies. Verteporfin directly targets and modifies p62 [39]. A previous study in a pancreatic cancer xenograft animal model showed that verteporfin causes autophagy inhibition and enhances antitumor activity [40]. Second, the mechanisms by which LSD1 ubiquitinizes p62 (Figure 4C) remain unclear. Although the C-terminus of p62 is a ubiquitin-associated domain (UBA) [27], the ubiquitination of p62 appears independent of direct p62 demethylation by LSD1 (Supplementary Figure 3).

We conclude that LSD1 is overexpressed and promotes tumorigenesis in gynecologic malignancies (ovarian cancer and USC). LSD1 destabilizes p62 and inhibits autophagy in malignant cells. LSD1 downregulation reduces cancer cell growth but also activates autophagy. Suppression of both LSD1 and p62 displays additive inhibitory effect on cancer cell viability. A better understanding of the role played by p62 will be required to shed more light on the anticancer effects of LSD1 inhibitors.

## MATERIALS AND METHODS

### Immunohistochemistry and clinical tissue specimens

This translational study was approved by the local Institutional Review Board (IRB No.101-4771B). Immunohistochemistry (IHC) was performed on a commercially available ovarian cancer tissue array (BC111110; US Biomax Inc, Rockville, MD, USA; Supplementary Table 1). Formalin-fixed paraffin-embedded (FFPE) USC specimens were not available in the tissue array and were therefore retrieved from our Tumor Bank (Supplementary Table 2). The methodology used for IHC has been previously described in detail [41–44]. In brief, FFPE sections (4-μm thick) were deparaffinized in xylene and rehydrated through a series of graded ethanol. Sections were stained with a rabbit anti-human LSD1 polyclonal antibody on an automated IHC stainer with a DAB Detection system (Ventana Medical Systems, Tucson, AZ, USA). Hematoxylin was used for counterstaining. The overall immunohistochemical score (termed histoscore) was calculated by multiplying the percentage of positive cells (0−100%) by the intensity of the staining (graded as follows: 0, negative; 1, weak; 2, moderate; and 3, strong) [42, 43].

### Cell culture

Uterine serous carcinoma ARK2 cells were obtained from Dr. Alessandro Santin (Yale University, School of Medicine, New Haven, CT, USA) [45]. Human ovarian cancer (TOV112D, TOV21G) and endometrial cancer RL95-2 cell lines were purchased from the American Type Culture Collection (Manassas, VA, USA). ARK2 cells were grown in RPMI-1640 medium containing 10% fetal bovine serum. TOV112D, TOV21G, and RL95-2 cells were cultured in Dulbecco’s modified Eagle’s medium supplemented with 10% fetal bovine serum and appropriate amounts of penicillin and streptomycin at 37°C with 5% CO_2_.

### Antibodies, reagents and plasmids

Rabbit monoclonal antibodies raised against LSD1, ATG7, di-methyl-histone H3 (Lys4), histone H3, ubiquitin, and PARP were purchased from Cell Signaling Technology (Danvers, MA, USA); the anti-GAPDH antibody was from Santa Cruz Biotechnology (Santa Cruz, CA, USA); anti-Flag and anti-HA monoclonal antibodies were from Sigma (St. Louis, MO, USA); the anti-p62 rabbit polyclonal antibody was from GeneTex (San Antonio, TX, USA); the anti-LC3 rabbit polyclonal antibody was from Novus Biologicals (Littleton, CO, USA); the anti-methylated lysine rabbit polyclonal antibody was from Abcam (Cambridge, MA, USA). The LSD1 inhibitor SP2509 was obtained by Medchemexpress (Monmouth Junction, NJ, USA). All chemicals including chloroquine were purchased from Sigma, unless otherwise indicated. GFP-LC3 was kindly provided by Dr. Jennifer Leppincott-Schwartz (National Institutes of Child Health and Human Development, Bethesda, MD, USA).

### p62 protein stability assay

Cells were transiently transfected either with specific siRNA targeting LSD1 or control siRNA for 72 h. At baseline (i.e., before siRNA transfection), CHX (25 μg/mL) was added to the culture medium. Protein lysates were prepared at baseline as well as at 1- and 2-hour post-treatment and subjected to western blot analysis.

### DNA constructs

The pLenti-LSD1 was kindly provided by Dr. Hua-Chien Chen (Chang Gung University, Taiwan). The HA-p62 expression plasmid was purchased from Addgene (Cambridge, MA, USA). To generate truncated LSD1 and p62 proteins for cell expression, an appropriate set of oligonucleotide primers was utilized. The pLenti-LSD1 expression plasmid was used as a template, and primers were designed for flag-LSD1 cloning, as follows: 5’-AGCTTCTAGAGGATCCACTAGT-3’ (sense), and 5’-AGCTTCTAGACTCGAGCGGCCG-3’ (antisense). The fragment was digested and inserted in either orientation into the XbaI site of pFlag-CMV-2 (Sigma). Deletion constructs were prepared using the following primers: 5’-AGCTTCTAGAGGATCCACTAGT-3’ (sense) and 5’-TCTAGATTAGGGATTCGCTTCCAACTC-3’ (antisense); 5’-AGCTTCTAGACCAAGTGATGTATATCTCT-3’ (sense) and 5’-AGCTTCTAGACTCGAGCGGCCG-3’ (antisense), respectively. The deletion constructs (N-terminal and C-terminal) of HA-p62 were obtained using the following primers: 5’-GGTGGAATTCTATGGCGT-3’ (sense) and 5’-GGTAGCGGCCGCGGATCACATTGGGGTGCAC-3’ (antisense); 5’-AGCTGAATTCTGCGATGGCTGCAATGGGC-3’ (sense) and 5’-GGGTAGCGGCCGCGCAAC-3’ (antisense), respectively.

### Western blot

Cells were harvested, washed twice in phosphate-buffered saline (PBS), and lysed in ice-cold RIPA lysis buffer [1% Triton X-100, 1% NP40, 0.1% SDS, 0.5% DOC, 20 mM Tris-HCl pH 7.4, 150 mM NaCl, cocktail protease inhibitor (Sigma) for 30 min. Lysates were boiled in 4× sample buffer dye (250 mM Tris-HCl, pH 6.8, 8% SDS, 0.1% bromophenol blue, 40% glycerol, 400 mM β-mercaptoethanol) and subsequently subjected to 10% sodium dodecyl sulfate polyacrylamide gel electrophoresis (SDS-PAGE). Separated proteins in SDS-PAGE were electrotransferred to a Hybond-PVDF membrane (Amersham Pharmacia Biotech/GE Healthcare, Piscataway, NJ, USA). Blots were probed with designated primary antibodies and appropriate secondary antibodies. Finally, immunobands were detected with an enhanced chemiluminescence reaction (ECL, Amersham Pharmacia Biotech).

### Quantitative real-time QPCR

Quantitative real-time QPCR (RT-QPCR) was performed in duplicates on RNA specimens prepared in independent experiments. All transcript levels were normalized to GAPDH expression of each sample. Primer sequences were as follows: p62, 5’-CACCTGTCTGAGGGCTTCTC-3’ (sense) and 5’- CACACTCTCCCCAACGTTCT-3’ (antisense); GAPDH, 5’-GGTATCGTGGAAGGACTCATGAC-3’ (sense), 5’-ATGCCAGTGAGCTTCCCGT-3’ (antisense). The PCR conditions were as follows: initial denaturation for 10 min at 95°C, followed by 45 cycles of 95°C for 15 s and 60°C for 1 min. All reactions were performed on an ABI PRISM 7900 HT instrument (Applied Biosystems, Foster City, CA, USA). A mean cycle of threshold (Ct) value for each duplicate measurement was calculated.

### Immunoprecipitation

After the cells were harvested and washed twice in ice-cold PBS, cell pellets were resuspended in ice-cold WCE lysis buffer (20 mM HEPES, 10% glycerol, 0.5% Triton X-100, 0.2 M sodium chloride, 1 mM EDTA, 1 mM EGTA and protease inhibitor cocktail) for 30 min and centrifuged at 12 000 rpm at 4°C for 30 min. Equal amounts of cell extract protein were incubated with the designated antibodies (2 μg) at 4°C for 2 h. Immune complexes were captured with protein G-sepharose (30 μL; Upstate Biotechnology, Lake Placid, NY, USA) for 2 h at 4°C under rotation. The protein G-antigen-antibody complexes were washed four times with WCE lysis buffer and boiled in 4× sample buffer dye (250 mM Tris-HCl, pH 6.8, 8% SDS, 0.1% bromophenol blue, 40% glycerol, 400 mM â-mercaptoethanol) for subsequent PAGE and western blot analyses.

### RNA interference procedures

Cells were transiently transfected either with specific siRNA targeting LSD1, p62, or control siRNA (Ambion, Austin, TX, USA) using Lipofectamine RNAiMAX (Invitrogen/Life Technologies, Carlsbad, CA, USA) according to the manufacturer’s protocol. In brief, the Lipofectamine RNAiMAX reagent was incubated with the Opti-MEM medium without phenol red (Invitrogen/Life Technologies) for 5 min at room temperature. Specific siRNA was added to Lipofectamine RNAiMAX mixture and incubated at room temperature for 30 min to form the transfection complex. Transfection mixtures were then added to cells in Opti-MEM medium. After 72 h, cells were harvested for subsequent PAGE and western blot analysis. The sequences of siRNA LSD1#1 were 5’-GGUCUUGGAGGGAAUCCU-3’ (sense) and 5’-UAGGAUUCCCUCCAAGACC-3’ (antisense), whereas the sequences of LSD1#2 were 5’-GAGCAAGAGUUUAACCGGU-3’ (sense) and 5’-ACCGGUUAAACUCUUGCUC-3’ (antisense). The sequences of siRNA for p62 were 5’- GGAGCACGGAGGGAAAAGA-3’ (sense) and 5’- UCUUUUCCCUCCGUGCUCC-3’ (antisense). The sequences of negative-control siRNA were 5’-UAACGACGCGACGACGUAA-3’ (sense) and 5’-UUACGUCGUCGCGUCGUUA-3’ (antisense).

### *In vivo* ubiquitination assay

Cells were transiently transfected either with specific siRNA targeting LSD1 or control siRNA for 72 h. Subsequently, cells were treated with the proteasome inhibitor MG132 (10 μM) for 24 h. Negative control experiments without the use of MG132 were run in parallel. After cell harvesting, pellets were resuspended in WCE buffer and analyzed with immunoprecipitation and immunoblotting as described above.

### Immunofluorescent microscopy of puncta formation in autophagy

After transient transfection with a green fluorescent protein-tagged LC3 (GFP-LC3) expression plasmid, cancer cells were cultured overnight on a chamber slide. After treatment with siRNA or pharmacological compounds, cells were fixed with 3.7% formaldehyde for 5 min and incubated in blocking buffer (5% normal goat serum in PBS) for 1 h to reduce nonspecific binding. Slides were mounted with a specific medium (0100-20; SouthernBiotech, Birmingham, AL, USA) and analyzed with the Leica TCS SP2 laser-scanning confocal system (Leica, Wetzlar, Germany). GFP-LC3 fluorescence was calculated by counting the number of GFP-positive cells exhibiting punctate GFP-LC3 [34].

### Cell proliferation assay

The trypan blue assay was used to assess cell viability. Cells were seeded in full medium at a density of 1 × 10^4^ cells per well. Thereafter, they were trypsinized, stained, and counted at three different time points (at 24, 48, and 72 h after seeding).

### Clonogenic assay

Cells were transiently transfected with either specific siRNA targeting LSD1 or control siRNA for 72 h. Thereafter, a total of 5,000 cells were seeded into 6-well dishes and maintained for 10 days to investigate their clonogenic capacity and their ability to form colonies. To this aim, cells were fixed with 12.5% acetic acid in 30% methanol and stained with Brilliant Blue R.

### Proximity ligation assay (PLA)

The protocol for deparafinization of paraffin-embedded ovarian cancer sections was similar to that used for immunohistochemistry [44]. After incubation for 1 h in blocking solution (Thermo Scientific, Walthma, MA, USA), slides were stained with a combination of anti-LSD1 (Cell Signaling Technology, Danvers, MA, USA), anti-p62 antibodies (GeneTex), or an IgG control antibody (Sigma). The procedure was performed using a Duolink *in situ* Red starter kit mouse/rabbit (Sigma) according to the manufacturer’s protocol. Slides were finally analyzed on a Leica TCS SP2 laser scanning confocal system (Leica Inc.).

### Cell viability assay

Cancer cells were transiently transfected with si-C or p62 siRNA (si-p62) for 48 h. Approximately 1 × 10^4^ cells were subsequently placed in each well of a 96-well culture plate for 24 h. For viability experiments, cancer cells in serum-free medium were treated with SP2509 or TCP for 24 h. For the colorimetric MTT assay, MTT (5 mg/mL, 25 μL) was added into each well containing treated cells. The supernatant was discarded after 4 h and DMSO (100 μL) was then added to each well; the mixture was shaken and measured at 570 nm using an ELISA reader scanning multi-well spectrophotometer (PerkinElmer VICTOR 2, Waltham, MA, USA).

### Animals and treatment

All animal procedures were reviewed and approved by the Animal Care Committee of the Chang Gung Memorial Hospital Institutional Review Board (2015102001). Female BALC/c nude mice were obtained from the National Laboratory Animal Center, Taiwan. ARK2 cells were harvested, washed, and resuspended in Hanks’ balanced salt solution (HBSS) at a final concentration of 10^7^ cells/mL. Tumors were established by subcutaneous injection of cell suspensions (100 μL) into the lateral hind leg of mice aged 6−8 weeks. After 20 days, animals were treated with designated regimens: SP2509 (0.5 mg per 100 μL) twice per week, chloroquine (5 mg per 100 μL) 5 days per week, the combination of both reagents, or vehicle as control. During the treatment course, tumor growth was monitored on a weekly basis. Tumor volumes (cm^3^) in tumor-bearing mice were determined with an *in vivo* assay for tumor mass. Upon completion of the experiments, tumors were excised and extracted for western blot analysis.

### Statistical analysis

The LSD1 histoscores in tumor and control tissues were compared with the Mann-Whitney *U* test. All calculations were performed using the SPSS 17.0 statistical package (SPSS Inc., Chicago, IL, USA). Two-tailed P values <0.05 were considered statistically significant.

## SUPPLEMENTARY MATERIALS FIGURES AND TABLES




