# Supplementary material for: Lysine-specific demethylase 1 (LSD1) destabilizes p62 and inhibits autophagy in gynecologic malignancies
Source: Oncotarget. 2017 Aug 10;8(43):74434–50. doi: 10.18632/oncotarget.20158 (PMC5650353; doi:10.18632/oncotarget.20158)
Supplement: Supplementary file 1 [file oncotarget-08-74434-s001.pdf]

Lysine-specific demethylase 1 (LSD1) destabilizes p62 and inhibits autophagy in gynecologic malignancies

SUPPLEMENTARY MATERIALS

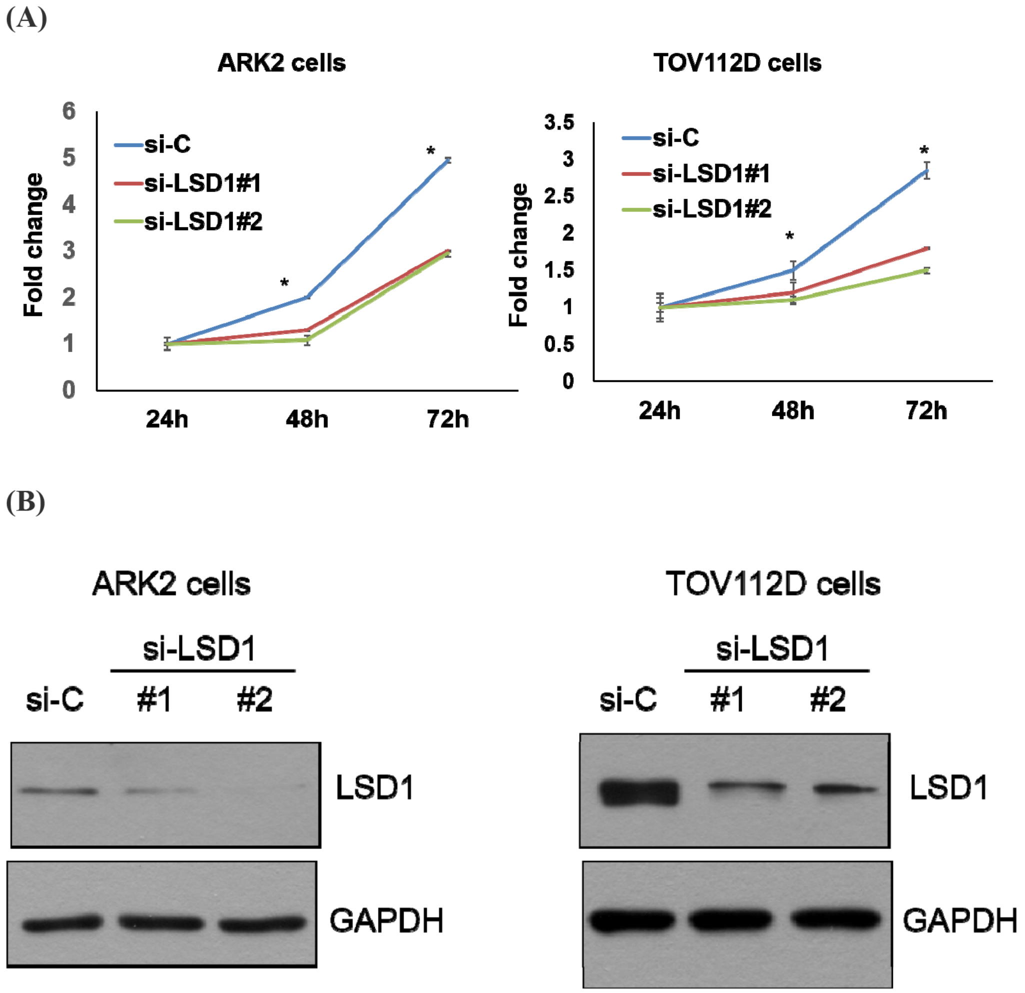

Supplementary Figure 1: (A) Uterine serous carcinoma ARK2 cells and ovarian carcinoma TOV112D cells were transiently transfected with control siRNA (si-C), LSD1 siRNA #1 or LSD1 siRNA #2 for 72h, knocking down LSD1 resulted in a significant time-dependent decrease in cell proliferation. Data are expressed as means ± standard errors from three independent experiments. * $P < 0.05$ compared to control. **(B)** Cancer cells were transiently transfected with control siRNA (si-C), LSD1 siRNA #1 or LSD1 siRNA #2 for 10 days. Cell lysates were subsequently subjected to western blots. Equal amount of protein lysates were analyzed with western blots with appropriate antibodies.

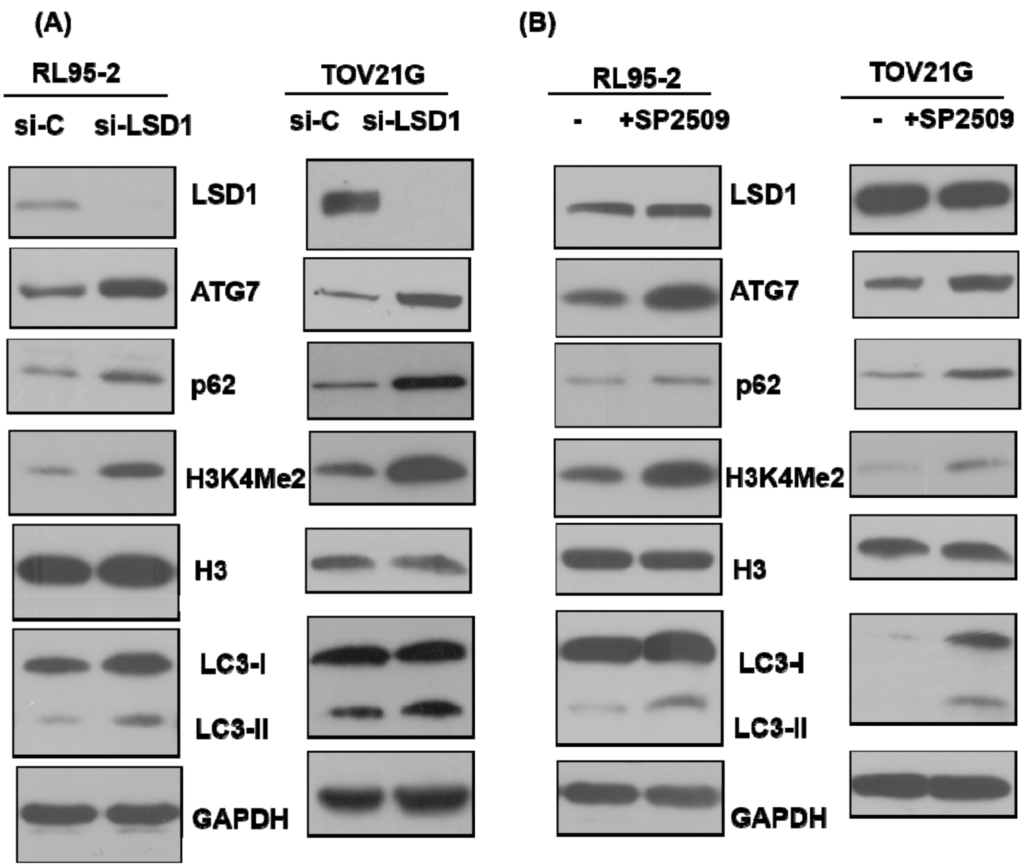

Supplementary Figure 2: Inhibition of LSD1 activates autophagy. **(A)** Endometrial cancer RL95-2 cells and ovarian cancer TOV21G cells were transiently transfected with si-C or LSD1 siRNA #2 for 72 h. **(B)** RL95-2 and TOV21G cells were treated with a LSD1 inhibitor SP2509 (100 nM) for 24 h. Equal amounts of protein lysates were subjected to immunoblotting with the designated antibodies. GAPDH was used to confirm equal protein inputs in all lanes.

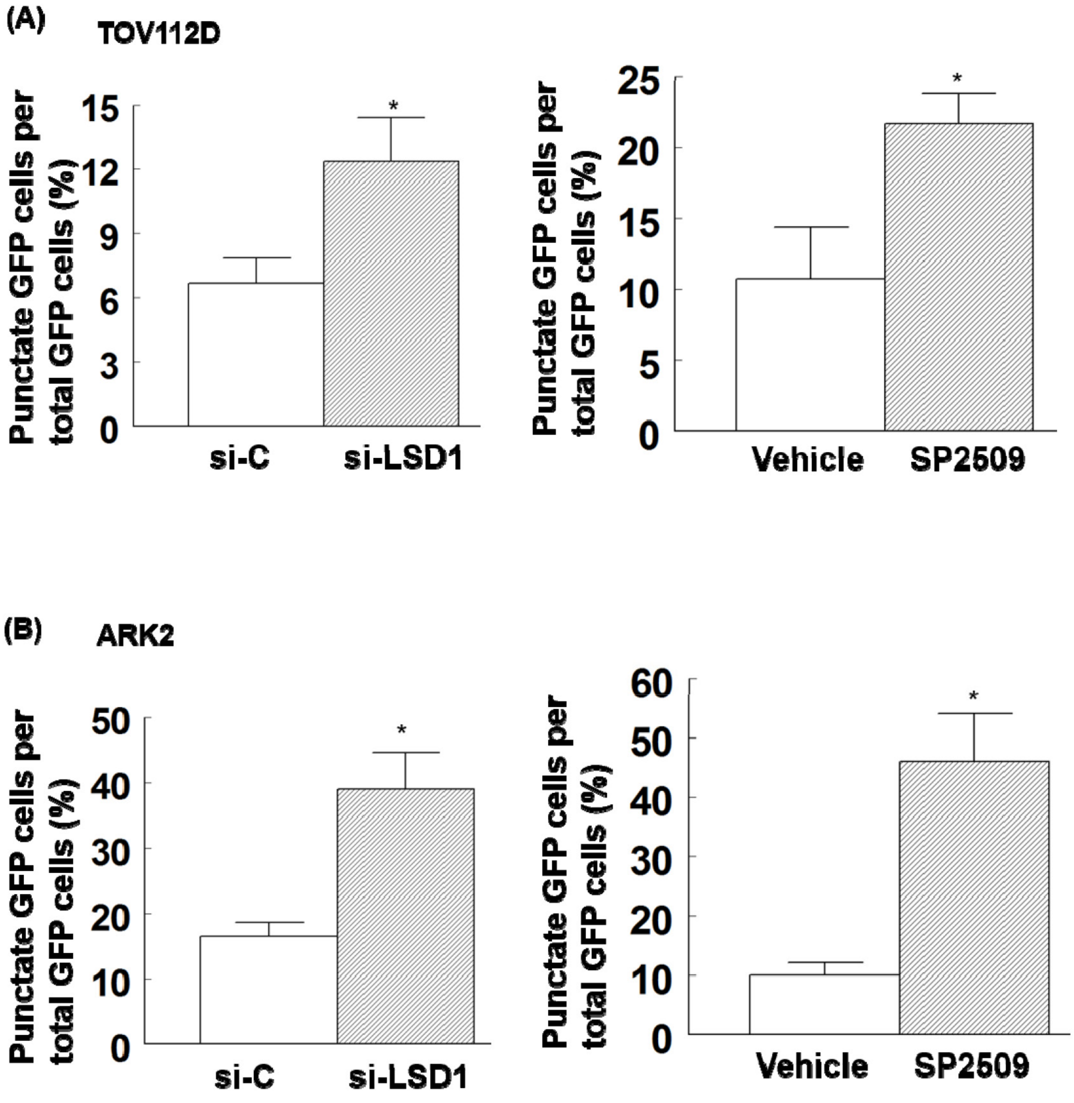

Supplementary Figure 3: Quantitative assessment of autophagy activated by LSD1 inhibition. Formation of immunofluorescent puncta structures were analyzed in **(A)** uterine serous carcinoma ARK2 cells and **(B)** ovarian cancer TOV112D cells that were transfected with GFP-LC3 and si-C or LSD1 siRNA #2 for 72 h or transfected with GFP-LC3 and treated with SP2509 for 24 h. Bar charts indicate the percentage of cells with formation of GFP-LC3 punctuate structures. * $P < 0.05$ compared to control.

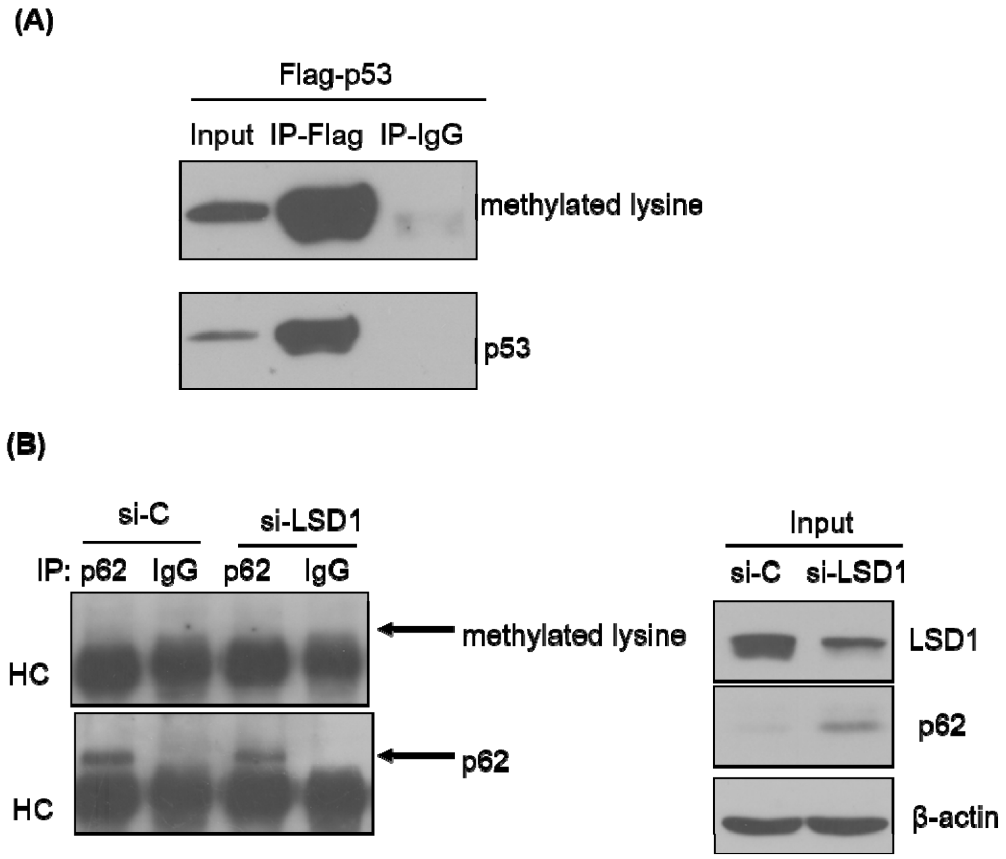

Supplementary Figure 4: LSD1 does not demethylate p62. **(A)** Uterine serous carcinoma ARK2 cell lysates transiently overexpressing Flag-tagged p53 (Flag-p53) were immunoprecipitated (IP) with an anti-Flag antibody and subsequently subjected to immunoblotting with antibodies raised against methylated lysine or p53. **(B)** ARK2 cells were transiently transfected with si-C or LSD1 siRNA #2 for 72 h, and the whole-cell lysates were immunoprecipitated (IP) with an anti-p62 antibody. They were subsequently analyzed by immunoblotting with antibodies raised against methylated lysine or p62. A control antibody (IgG) was used for mock immunoprecipitation. The immunoblotting of input proteins (1/50 of the IP) is shown on the right panel of (B). HC: IgG heavy chain.

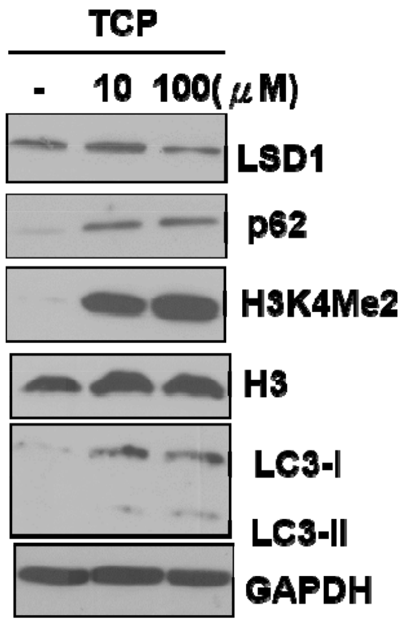

Supplementary Figure 5: Inhibition of LSD1 increases p62 levels and activates autophagy. Uterine serous carcinoma ARK2 cells were treated with 10 µM or 100 µM of TCP for 24 h. Equal amounts of protein lysates were separated by SDS-PAGE and subjected to immunoblotting with the indicated antibodies. GAPDH was used to confirm equal protein inputs in all lanes.

Supplementary Table 1: Ovarian cancer tissue array (BC111110; US Biomax Inc, Rockville, MD, USA) for immunohistochemical analysis

See Supplementary File 1

Supplementary Table 2: Formalin-fixed paraffin-embedded (FFPE) of uterine serous carcinomas of endometrium specimens for immunohistochemical analysis

ID	Age	FIGO stage	Grade	Cell type
1	46	1	1	serous
2	42	3	3	serous
3	57	1	2	serous
4	63	1	3	serous
5	75	1	3	serous
6	65	3	3	serous
7	61	3	3	serous
8	51	3	3	serous
9	71	4	3	serous
10	53	4	3	serous
11	57	1	3	serous
12	61	1	3	serous
13	61	1	3	serous
14	59	2	3	serous
15	55	1	2	serous
16	66	1	2	serous
17	65	4	3	serous
18	53	4	3	serous
19	68	3	3	serous
20	60	1	2	serous
21	56	1	3	serous
22	75	3	3	serous
